# The Interprofessional Education Exchange: The Impact of a Faculty Development Program in Interprofessional Palliative Oncology Education on Trainee Competencies, Skills, and Satisfaction

**DOI:** 10.1089/pmr.2021.0045

**Published:** 2021-10-27

**Authors:** Tara J. Schapmire, Barbara A. Head, Christian D. Furman, Carol Jones, Bonika Peters, M. Ann Shaw, Frank Woggon, Craig Ziegler, Mark P. Pfeifer

**Affiliations:** ^1^ Division of General Internal Medicine, Palliative Care and Medical Education, University of Louisville School of Medicine, Louisville, Kentucky, USA.; ^2^ Kent School of Social Work, University of Louisville, Louisville, Kentucky, USA.; ^3^ Trager Institute, University of Louisville, Louisville, Kentucky, USA.; ^4^ Department of Family and Geriatric Medicine, University of Louisville School of Medicine, Louisville, Kentucky, USA.

**Keywords:** collaborative practice education, faculty development, interprofessional curriculum, interprofessional education, oncology, palliative care

## Abstract

***Background:*** The interprofessional education exchange (iPEX) provides education, training, and mentoring to select interprofessional faculty trainee teams for development and implementation of interprofessional education (IPE) in palliative oncology.

***Objective:*** To evaluate the impact of the iPEX project on trainees' self-efficacy in IPE skills and IPE competencies.

***Design:*** A pre-/post-test design was used to evaluate trainees' progress. Trainees rated project components and developed IPE curricula in palliative oncology.

***Setting/Subjects:*** Sixteen United States-based faculty teams consisting of four to five members representing three or more disciplines completed the one-year faculty development project consisting of webinars, online interactive modules, a face-to-face workshop, mentoring, and assistance. The exchange of ideas, means for overcoming obstacles, collaborative teaching techniques, and curriculum development guidelines were integrated into the program.

***Measurements:*** Standardized measures of self-efficacy in IPE skills (Interprofessional Facilitation Skills Checklist) and IPE competencies (Core Competencies for Interprofessional Practice Individual Competency Assessment Tool) were used. Trainees rated the effectiveness of the project components on a scale of 1–5 (1 = not at all effective, 5 = extremely effective) and reported their plan for IPE palliative care curricula at their home institution.

***Results:*** Pre and post-paired samples *t*-test scores (*n* = 78) on both standardized instruments for IPE skills and competencies were significantly different (*p* < 0.001). Ratings of project components ranged from 3.97 to 4.90. Each team successfully developed a unique plan for IPE in palliative oncology.

***Conclusions:*** Multimodal faculty development and mentoring are successful means for improving self-assessed IPE skills and competencies.

## Background

### Importance of interprofessional education

The need to educate health professional students collaboratively has long been cited as a way to improve patient outcomes, particularly those related to quality and safety.^1,2^ Models of care foundational to health care reform mandate team-based patient-centered collaborative care. Numerous studies of health care education conducted by the National Academies of Sciences, Engineering, and Medicine have concluded that interprofessional education (IPE) is essential to prepare future practitioners to work effectively in present health care environments.^3–6^ The Institute of Medicine report, Dying in America,^7^ states that “educational silos that impede the development of interprofessional teams limit better palliative care” and recommends that “all clinicians across disciplines and specialties who care for people with advanced serious illness should be competent in basic palliative care, including communication skills, interprofessional collaboration, and symptom management”. The report adds that accrediting organizations should require palliative care education for all specialties. In 2009, six health care educational associations partnered to form the interprofessional education collaborative (IPEC) and jointly established core competencies for interprofessional collaborative practice to guide IPE efforts.^8^ In addition, the World Health Organization's report, Framework for Action on Interprofessional Education and Collaborative Practice, claimed that IPE and practice was necessary across the globe and offered strategies for implementing IPE.^9^ An analysis of accreditation documents for 10 health professions identified 60 statements significant to IPE.^10^

### Palliative care and IPE

Palliative care training has been strongly endorsed for all health science learners.^11^ Palliative care is becoming an essential component of health care throughout the world, making it critical that those entering health professions understand the principles and practices of quality palliative care across the disease continuum.^12–14^ A core tenet of palliative care is interdisciplinary team-based collaborative care.^15^ Using palliative oncology care as the framework for teaching interprofessional competencies combines essential training in oncology care, palliative care, and team-based practice.

### Faculty development for IPE

Unfortunately, most health professional education still occurs in discipline-specific silos with minimal interaction among professions. Competencies, strategies, and accreditation requirements for IPE are clearly outlined^8,9^; however, developing and implementing IPE activities at the grass roots level challenge institutions. Crowded curricula, differing academic calendars, disparate cultures between disciplines, and logistical issues are some barriers that may seem insurmountable.^16^ Reviews of the literature have found that IPE faculty development programs can contribute to the progression and success of IPE for health professions.^17,18^

The purpose of this study is to evaluate the impact of the National Institutes of Health (NIH)-funded interprofessional education exchange (iPEX) faculty development project on IPE competencies and skills and the development of IPE palliative care curricula. This article reports the results of the faculty development component of the project; a future article will report on the details of the curriculum projects created and implemented, as well as students impacted.

## Methods

A pre-/post-test design was used to evaluate trainees' progress related to IPE competencies and skills. Trainees rated project components and developed IPE curricula in palliative oncology. Trainee teams reported on their tailored plans for IPE in palliative oncology at their home institutions.

### Participants

Participant faculty teams were selected using a competitive process. Each team had to have representatives from at least three health professions, three to five faculty trainees per team, at least one member with responsibility for curriculum planning or experienced in interprofessional relationship development, documented financial and institutional support for project participation, and appropriate school or program accreditation. Applications provided background information on each team member and initial thoughts about a plan for such curricula at their home institution. The iPEX core faculty scored applications. Eight to nine teams were selected annually.

### The curriculum

The foundational framework for the iPEX activities focused on the two target audiences: institutions and faculty teams (the targeted trainees). On the institutional level, participant site teams will use performance improvement and the plan-do-study-act approach to plan, act, and evaluate their involvement. During the plan phase, the faculty teams assembled and clearly defined what they hoped to accomplish. A strength-weaknesses-opportunities-threats (SWOT) analysis was completed to examine the institution's current situation related to IPE and cancer education efforts. Specific goals, timelines, and flowcharts were developed. Faculty teams were involved in webinars and completion of planning instruments to facilitate the initial institution-specific planning that took place as a first step toward curriculum implementation. The do phase involved teams attending the workshops to develop skills, design their curriculum, and develop their curriculum implementation plan. Mentor feedback and process evaluation occurred during the study phase. With the help of experienced mentors, faculty teams identified weaknesses and opportunities for improvement as they planned for and implemented an IPE curriculum teaching palliative care in oncology at their site. During the act phase, the faculty teams continued to examine and re-examine processes and implement adjusted approaches as needed.

At the individual level, knowing that participating faculty members were self-directed, motivated, and experienced, teaching/learning approaches directed toward this group were based upon the andragogical theory of adult learning.^19^ In addition, the design of the iPEX training incorporated diffusion of innovations theory,^20,21^ which has been used for decades to understand steps and processes required to achieve dissemination and diffusion of knowledge. In the preworkshop phase including a SWOT analysis and needs assessment, faculty teams were encouraged to explore the potential for innovation diffusion success at their institutions by examining the variables that can explain variability in successful outcomes: (1) characteristics of the innovation, (2) characteristics of the adopters, and (3) features of the setting or environmental context. Over the course of the project, faculty teams were supported through all the stages of diffusion, from innovation development, adoption, and implementation, thru maintenance, sustainability, and institutionalization.

**FIG. 1.**
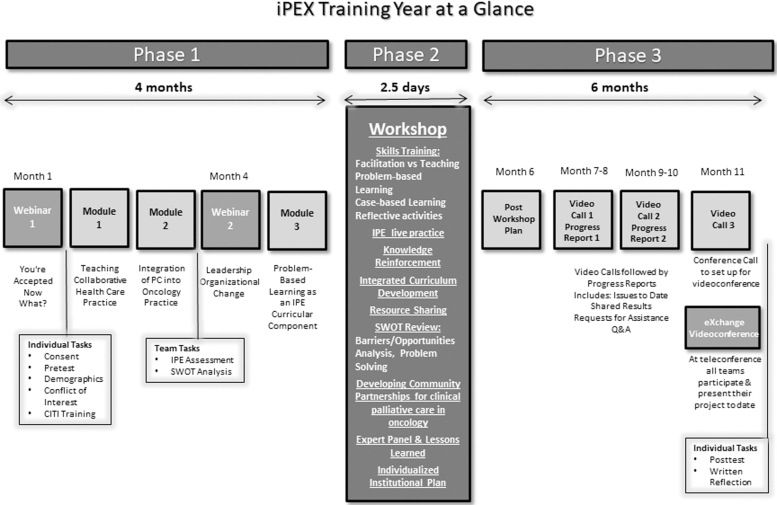
Training year at a glance.

The multimodal one-year curriculum was designed from the outset with the intent to model the very skills and competencies we hoped to improve in our faculty trainees—from collaborative teaching approaches to problem-based learning—and provided (1) a skills training program including faculty development and institutional analysis, (2) resources, including an established evidence-based tested curriculum,^16,22^ and (3) expert planning and implementation guidance. The focus was not on teaching palliative care, but on how to collaboratively teach collaborative and integrated palliative care in interprofessional settings. The training project consisted of three phases as described hereunder (Fig. 1):

#### Phase 1: Trainee preparation

Preparation for the workshop began with an introductory webinar (*You're Accepted—Now What?*) outlining the project and orienting trainees to the website. Trainees then completed self and institutional assessments. The results of these evaluations and means for overcoming IPE barriers and challenges were discussed during a second webinar (*Leadership and Organizational Change*). Trainees completed three interactive online modules: *Teaching Collaborative Health Care Practice, Integration of Palliative Care into Oncology Practice,* and *Problem-Based Learning as an IPE Curricular Component.*

A core faculty liaison was assigned to each team. This liaison was an experienced educator, well versed in IPE. The liaison guided the team through project activities and was available to answer questions, make suggestions, and connect teams with needed resources.

#### Phase 2: Workshop

A two and a half day face-to-face workshop was held at a central location four months into the project. The focus of the workshop was hands-on skills training, exchange of ideas, and the development of a team plan for IPE in palliative oncology tailored to the context and needs of their home institution. Trainees worked together to develop solutions to obstacles they face. Trainees practiced skills such as curriculum development, team building, collaborative learning techniques, group facilitation, analysis of group process, development and response to reflective activities, addressing challenging situations, and evaluating student outcomes.

Teams were given time to work on an IPE plan for their home institution with guidance from their iPEX faculty liaison. At the end of the workshop, each team presented its plans and received feedback from the entire group. Opportunities to exchange ideas and experiences were integrated throughout the workshop.

#### Phase 3: Site implementation with ongoing support

After the workshop, the team liaison continued mentoring the team in plan development and implementation. Progress reports were submitted every other month. On the alternate months, the liaison conducted a video call with the team to review progress, assisted with troubleshooting, connected the team to resources, and monitored the ongoing plan. Formal mentoring and support culminated in the exchange video conference, in month 11, where teams shared plans and progress. The iPEX project continued providing support and assistance for two years after the training. Figure 1 outlines the curriculum plan.

### Instruments

Two standardized instruments were used in the pre and post-test questionnaire.

#### Faculty Interprofessional Facilitation Skills Scale^23^

This scale, developed from a validated framework of competencies for interprofessional facilitation, assesses performance on IPE skills and has been shown to have high internal consistency. The Interprofessional Facilitation Skills (IPFS) instrument addresses the trainees' self-perceived skill level related to two domains: contextualize IPE and encourage interprofessional interaction. The composite scores are calculated by averaging the score of items that make up each domain.

#### Core competencies for interprofessional practice individual competency assessment tool (IPEC)^24,25^

This instrument assesses competencies related to four competency domains for interprofessional collaborative practice developed by the IPEC: value and ethics, teams and teamwork, roles and responsibilities, and interpersonal communications. Trainees rate their competency levels from novice to expert on 39 individual competencies.

The utility and quality of the workshop, online modules, calls with liaisons, the website, email reminders and updates, the webinars, and progress reports were also evaluated on a Likert scale.

The internal consistency of the four IPEC scales and the two IPFS scales for this population was evaluated by Cronbach's alpha. For the prescales, Cronbach's alpha ranged from 0.82 (IPFS Context IPE) to 0.97 (IPEC team and teamwork). Five of the scales Cronbach's alpha were considered excellent (≥0.95). For the postscales, Cronbach's alpha ranged from 0.72 (IPFS Context IPE) to 0.96 (IPEC team and teamwork), where five of the scales Cronbach's alpha were again excellent (≥0.90).

Teams reported on their plan for IPE in their home institutions and on progress made as indicators of the desired project outcome (an established IPE curriculum in oncology palliative care).

This study was reviewed and approved by the university's institutional review board.

### Statistical analysis

Demographic data were tabulated, and frequencies, percentages, means, and standard deviations are presented where appropriate. Paired sample *t* test compared the pre and post outcomes for the four composite IPEC scales and the two composite IPFS scales, whereas a Welch one-way analysis of variance (ANOVA) was performed to assess differences on the change score among groups. The Welch's ANOVA was performed because this analysis is believed to address disproportional group sizes most effectively and does not assume equal variances among groups.^26,27^

Pre and post difference effect sizes are presented by Cohen's D. Cohen describes an effect size of 0.20 to 0.49 as small, 0.50 to 0.79 as moderate, and ≥0.80 as large.

For the survey items that evaluated the components of the training, the data were tabulated, and frequency, percentages, means, and standard deviations are reported. For all analyses, statistical significance was set by convention at *p* < 0.05. SPSS version 26.0^28^ was used to analyze the quantitative data.

## Results

### Demographics

Seventy-eight trainees participated over the two-year period. All completed the pre and post surveys making the response rate 100%. Team members were predominantly women (76%) and white (88%). Nursing (29%), medicine (21%), and social work (19%) were the top three disciplines that participated in the training. See Table 1 for complete demographics.

**Table 1.** Demographics

**Characteristic**	***n***	**%**
Gender		
Male	18	23
Female	59	76
Other/prefer not to report	1	1
Race		
American Indian/Alaska Native	0	0
Asian	3	4
Native Hawaiian or other Pacific Islander	0	0
Black or African American	3	4
White	69	88
More than one race	2	3
Other/prefer not to report	1	1
Ethnicity		
Hispanic or Latino	6	8
Not Hispanic or Latino	67	86
Other/prefer not to report	5	6
Age, mean (SD)	45.6 (10.1)	
Discipline		
Chaplaincy	4	5
Dentistry	1	1
Medicine	16	21
Nursing	23	29
Optometry	1	1
Pharmacy	6	8
Physical therapy	2	3
Psychology	1	1
Social work	15	19
Speech therapy	1	1
Other	8	10
Type of discipline		
Medical	52	67
Psychosocial	26	33
Experience in interprofessional education		
None	8	10
Limited (one year or less)	22	29
Moderate (two to three years)	16	21
Significant (more than three years)	31	40
Prefer not to report	1	
Cohort		
One	38	49
Two	40	51

SD, standard deviation.

### Instruments

All domains of the IPEC and the IPFS subscales (Table 2) showed significant pre to post-test differences for trainees, *p* < 0.001, with the effect size for improvement being large for all domains (Cohen's D ranging from 0.79 to 1.19).

**Table 2.** Analysis of Pre and Post Mean Interprofessional Education Collaborative Competencies and Interprofessional Facilitation Skills Scales on All Data

		**Pre**	**Post**	***p*** ^**a**^	**Cohen's D**
	**Count**	**Mean (SD)**	**Mean (SD)**		
IPEC					
Value/ethics: (range 1–10)	78	7.66 (1.34)	8.65 (0.83)	<0.001	0.79
Team and teamwork: (range 1–10)	78	6.20 (1.79)	7.90 (1.12)	<0.001	1.06
Roles/responsibilities: (range 1–10)	77	7.03 (1.36)	8.35 (0.91)	<0.001	1.06
Interpersonal communication: (range 1–10)	77	7.05 (1.43)	8.25 (0.92)	<0.001	1.00
IPFS					
Contextualize IPE (range 1–4)	78	3.12 (0.55)	3.71 (0.37)	<0.001	1.02
Encourage interprofessional interaction (range 1–4)	78	2.94 (0.52)	3.62 (0.37)	<0.001	1.19

^a^ *p*-Value based on paired sample *t*-test.

Cohen's D reflects the effect size of the pre and post change where 0.20 to 0.49 is a small effect, 0.50 to 0.79 is a moderate effect, and ≥0.80 is a large effect.

IPE, interprofessional education; IPEC, interprofessional education collaborative; IPFS, interprofessional facilitation skills.

An analysis was performed on both the IPEC domains and the IPFS domains among five different discipline trainee groups to assess whether differences within the groups occurred going from pre to post-training and whether difference occurred among groups on their change scores (Table 3).

**Table 3.** Analysis of Pre and Post Mean Interprofessional Education Collaborative Competencies and Interprofessional Facilitation Skills Scales by Discipline (Chaplaincy, Medicine, Nursing, Pharmacy, and Social Work)

	**Count**	**Pre** **Mean (SD)**	**Post** **Mean (SD)**	***p*** ^**a**^	**Cohens D for change score**	**Change score mean (SD)**	***p*** ^**b**^
IPEC							
Value/ethics: (range 1–10)							
Chaplaincy	4	6.68 (1.02)	9.05 (0.56)	0.053	1.56	2.38 (1.53)	0.307
Medicine	16	7.65 (0.88)	8.49 (0.59)	**0.005**	1.17	0.84 (0.72)	
Nursing	23	8.03 (1.22)	8.58 (1.03)	0.053	0.43	0.55 (1.29)	
Pharmacy	6	7.86 (1.10)	8.75 (1.09)	**0.023**	1.51	0.89 (0.59)	
Social work	15	7.61 (1.34)	8.72 (0.79)	**0.005**	0.98	1.11 (1.13)	
Team and teamwork: (range 1–10)							
Chaplaincy	4	4.91 (0.88)	8.43 (0.84)	**0.005**	4.19	3.52^c^ (0.84)	**0.010**
Medicine	16	6.10 (1.69)	7.96 (0.92)	**0.005**	1.18	1.86 (1.58)	
Nursing	23	6.69 (1.69)	7.81 (1.28)	**0.005**	0.72	1.12^c^ (1.55)	
Pharmacy	6	6.50 (1.38)	8.06 (1.54)	**0.021**	1.36	1.56 (1.15)	
Social work	15	6.12 (2.12)	7.84 (1.23)	**0.005**	0.91	1.72 (1.88)	
Roles/responsibilities: (range 1–10)							
Chaplaincy	4	6.53 (0.53)	9.00 (0.74)	**0.010**	3.17	2.47 (0.78)	**0.050**
Medicine	16	7.03 (1.32)	8.42 (0.67)	**0.003**	1.14	1.39 (1.22)	
Nursing	23	7.49 (1.13)	8.31 (1.00)	**0.003**	0.8	0.83 (1.04)	
Pharmacy	6	7.25 (1.12)	8.62 (1.12)	**0.017**	1.44	1.37 (0.95)	
Social work	14	6.95 (1.38)	8.16 (1.01)	**0.005**	0.98	1.21 (1.23)	
Interpersonal communication: (range 1–10)							
Chaplaincy	4	6.59 (0.46)	8.63 (0.71)	0.090	2.31	2.03 (0.88)	0.300
Medicine	16	7.01 (1.43)	8.25 (0.67)	**0.003**	1.08	1.24 (1.15)	
Nursing	23	7.27 (1.12)	8.22 (1.03)	**0.003**	0.93	0.95 (1.02)	
Pharmacy	6	6.79 (1.62)	8.38 (1.39)	**0.010**	1.72	1.58 (0.92)	
Social work	14	7.04 (1.54)	8.16 (1.07)	**0.008**	0.90	1.13 (1.26)	
IPFS							
Contextualize IPE (range 1–4)							
Chaplaincy	4	2.92 (0.63)	3.75 (0.32)	0.194	0.94	0.83 (0.88)	0.601
Medicine	16	3.17 (0.47)	3.54 (0.40)	**0.023**	0.69	0.37 (0.54)	
Nursing	23	3.14 (0.51)	3.78 (0.37)	**0.003**	1.25	0.64 (0.51)	
Pharmacy	6	3.50 (0.69)	3.89 (0.17)	0.287	0.49	0.39 (0.80)	
Social work	15	3.04 (0.63)	3.67 (0.40)	**0.003**	1.13	0.62 (0.55)	
Encourage interprofessional interaction (range 1–4)							
Chaplaincy	4	3.06 (0.56)	3.79 (0.25)	**0.034**	1.87	0.73 (0.39)	0.759
Medicine	16	2.88 (0.49)	3.41 (0.39)	**0.008**	0.80	0.53 (0.66)	
Nursing	22	2.93 (0.54)	3.60 (0.42)	**0.003**	1.1	0.67 (0.61)	
Pharmacy	6	3.04 (0.55)	3.76 (0.23)	**0.034**	1.24	0.72 (0.58)	
Social work	14	2.83 (0.57)	3.68 (0.38)	**0.003**	1.47	0.85 (0.58)	

Bolded values are statistically significant values.

^a^ *p*-Value based on paired sample *t*-test with the Benjamini–Hochberg *post hoc* correction applied.

Cohen's D reflects the effect size of the pre and post change where 0.20 to 0.49 is consider a small effect, 0.50 to 0.79 is a moderate effect, and ≥0.80 is a large effect.

^b^ *p*-Value reflects the Welch ANOVA test to determine whether the pre to post change scores for the five disciplines differed.

^c^ Matching superscripted letters reflects significant differences in the discipline groups change score based on the Games Howell *post hoc* test.

ANOVA, analysis of variance.

For the IPEC values and ethics and the interpersonal communication domains, significance was not found among disciplines on the change scores. For the values and ethics domain, the disciplines of medicine, pharmacy, and social work had significant improvements going from pre to post-training, *p* < 0.05. For chaplaincy and nursing, significant improvements were not found, *p* = 0.053. The effect sizes for trainees in chaplaincy, medicine, social work, and pharmacy were large (Cohen's D ≥ 0.98); nurses had a small-to-moderate effect, (Cohen's D = 0.43). The domains of teams and teamwork and roles and responsibilities for each discipline had significant improvements going from pre to post-training, *p* < 0.05, with all effect sizes large for all disciplines (Cohen's D ≥ 0.08), except for nurses in the team and teamwork domain having a moderate effect (Cohen's D = 0.72). For teams and teamwork, a significant difference was found between chaplains and nurses on their change scores. The IPEC interpersonal communication domain revealed all training disciplines experienced a large effect (Cohen's D ≥ 0.90), however, chaplains were the only group that did not achieve significant pre to post-training improvements, *p* = 0.190.

For the IPFS domains, significance was also not found on the change scores comparisons among groups. For the contextualize IPE domain, significant improvements with moderate-to-large effects were found for medical, nursing, and social work trainees (Cohen's D ≥ 0.69). Significant improvements were not found for chaplaincy or pharmacy trainees. For the encourage IP interaction domain, all disciplines had significant improvements and large effects sizes, Cohen's D ≥ 0.80.

To assess whether the fidelity of the training was standardized, an analysis was performed to assess whether differences were found between groups in cohort 1 (year 2019) and cohort 2 (year 2020). The analysis showed that there were no significant differences in change scores for any of the IPEC or IPFS domains. Furthermore, there were no significant differences on the prescore domains or the postscore domains by cohorts, *p* > 0.05 (data not shown).

### Training evaluation

The post-test evaluation of the training showed many components of the workshop were rated highly in terms of usefulness and quality. Overall, 100% of participants rated the workshop useful or very useful and 99% rated the quality of the project as well done or excellent. The mean score on rating five of the training components (workshop, online modules, calls with liaisons, email reminders and updates, and website) as useful was >4.0 on a 1 to 5 response format scale (Table 4). Similarly, the quality of all workshop components (workshop, liaison relationships, responses to inquiries, explanation of the project requirements, modules, website, application process, recruitment process, and webinars) had mean scores >4 on a 1 to 5 response format scale (Table 5).

**Table 4.** Usefulness of the Interprofessional Education Exchange Program Components

	**Not useful at all (1)**	**Not very useful (2)**	**Neutral (3)**	**Useful (4)**	**Very useful (5)**	
	***n* (%)**	***n* (%)**	***n* (%)**	***n* (%)**	***n* (%)**	**Mean (SD)**
Workshop	0 (0)	0 (0)	0 (0)	8 (10)	70 (90)	4.90 (0.31)
Online modules	0 (0)	3 (4)	7 (9)	39 (51)	27 (36)	4.18 (0.76)
Calls with liaisons	2 (3)	0 (0)	13 (17)	30 (38)	33 (42)	4.18 (0.89)
Email reminders and updates	0 (0)	3 (4)	8 (10)	32 (41)	35 (45)	4.27 (0.80)
Website	1 (1)	0 (0)	15 (19)	30 (39)	31 (40)	4.17 (0.83)
Webinars	0 (0)	1 (1)	16 (21)	44 (57)	16 (21)	3.97 (0.69)
Progress reports	0 (0)	3 (4)	19 (25)	33 (43)	22 (29)	3.96 (0.83)

**Table 5.** Quality of the Interprofessional Education Exchange Program Components

	**Poor (1)**		**Below average (2)**		**Neutral (3)**		**Well done (4)**		**Excellent (5)**			
	**Count**	**%**	**Count**	**%**	**Count**	**%**	**Count**	**%**	**Count**	**%**	**Mean (SD)**	
Workshop	1	1	0	0	0	0	8	11	67	88	4.84	(0.54)
Liaison relationship	0	0	1	1	5	6	12	16	59	77	4.68	(0.66)
Responses to inquiries	0	0	0	0	5	6	18	23	55	71	4.64	(0.60)
Explanation of the program requirements	1	1	1	1	4	5	34	44	38	49	4.37	(0.76)
Modules	0	0	2	3	5	6	36	47	34	44	4.32	(0.72)
Website	0	0	0	0	11	15	34	45	30	40	4.25	(0.70)
Application process	0	0	0	0	10	13	42	55	25	32	4.19	(0.65)
Recruitment process	0	0	1	1	13	17	41	53	22	29	4.09	(0.71)
Webinars	0	0	0	0	12	15	49	63	17	22	4.06	(0.61)

## Discussion

We report favorable outcomes in the implementation and evaluation of a national training program providing IPE-specific faculty development for faculty teams from institutions across the United States. The multimodal iPEX training program proved feasible. The 78 individuals completing the faculty development program showed significant pre to post-training improvement in IPE skills and self-competency with domains including teams and teamwork, values and ethics, and interpersonal interactions. Significant growth from pre- to post-test occurred for each discipline, on all subscales of the IPEC and IPFS, except for values/ethics for chaplains and nurses (nurses' pretest score was initially high), interpersonal communication for chaplains, and contextualize IPE and roles and responsibilities for chaplains and pharmacy. The lack of significance for chaplaincy and pharmacy likely resulted from a low sample size (*n* = 4), which may lead to lower statistical power. Participants found the experience to be highly useful with high-quality program components.

Importantly, these results occurred among faculty from varied institutional structures and wide-ranging geography, as well as disparate experience in IPE. This diversity reinforced the importance of developing tailored IPE programs rather than using standardized approaches.

The iPEX faculty development program was feasible for busy professionals. Key elements allowing full participation included using on-line methods for most content delivery, implementing only time-efficient and unique value-added curricular components, and using the three-day workshop exclusively for skills training and team program development. Chicago was a central hub for efficient travel. Interest in this training approach was high. For each cycle, applications far exceeded available capacity.

Lessons learned during implementation were numerous. First, the value and need for team time to work together in a mentored environment led to adjustments in the workshop program. In addition, although we created events for sharing ideas, we underestimated the value of this exchange for program development. The exchange environment of eight teams at each workshop and the year-end joint video presentations helped teams collaborate, find solutions to common barriers to IPE, and expand ideas and approaches. Finally, after completing most of their program design at the workshop, programs were best supported by regularly scheduled individual mentor meetings rather than combined national videoconferencing.

There are several limitations to these findings. Half of the participants were nurses or physicians. The smaller number of individuals in other fields such as chaplaincy and pharmacy might have impacted statistical significance despite strong effect sizes. Importantly, participants were self-selected, desiring additional training and program development. The methods and structure were effective for this cohort but cannot be assumed effective with less enthusiastic participants. The outcomes measured were self-assessed by trainees. Finally, the impact of this training and program development will require many years of observation to prove sustainable impact.

The very nature of the care of those with advanced illness will require continuous improvement in interprofessional practice and education. To promote these advances, additional training opportunities and the dissemination of results from multiple effective program designs are essential.

## Conclusion

The iPEX project improved self-assessed IPE skills and self-competency in teams and teamwork, roles and responsibilities, values and ethics, and interpersonal interactions. The training was perceived as valuable and useful and resulted in tailored plans for palliative care IPE at each participating institution.

## Ethical Approval

The treatment of all human subjects in this study was in accordance with the ethical standards of the University of Louisville Human Subjects Protection Program.

## Abbreviations Used

ANOVA: analysis of variance
IPE: interprofessional education
IPEC: interprofessional education collaborative
iPEX: interprofessional education exchange
IPFS: Interprofessional Facilitation Skills
NIH: National Institutes of Health
SD: standard deviation
SWOT: strength-weaknesses-opportunities-threats
